# Surface distributed acoustic sensing for mineral exploration

**DOI:** 10.1038/s41598-025-29964-6

**Published:** 2025-12-09

**Authors:** Lea Gyger, Alireza Malehmir, Musa Manzi, Oleg Valishin, Ronne Hamerslag

**Affiliations:** 1 Department of Earth Sciences, Uppsala University, Uppsala, Sweden; 2 School of Geosciences, University of the Witwatersrand, Johannesburg, South Africa; 3 Sercel, Carquefou, France; 4 Nordic Iron, Ludvika, Sweden

**Keywords:** Distributed acoustic sensing, Mineral exploration, Hardrock, Reflection seismology, Engineering, Natural hazards, Solid Earth sciences

## Abstract

As a receiver array for active-source seismic studies, the use of distributed acoustic sensing (DAS) technology is well established for vertical seismic profiling. However, given the sensitivity of fiber-optic cables, successful and cost-effective surface array studies in reflection seismology and for mineral exploration are still missing. In a feasibility study, we investigate the performance of surface DAS technology for mineral exploration in a hardrock environment. The target is an iron-oxide deposit located in the Bergslagen district in central Sweden. The fiber-optic cable was laid on the surface above the dipping mineralization and covered with a few centimeters of gravel to increase its ground coupling. The data quality varies significantly along the fiber cable. Nonetheless, it is possible to delineate the mineralization in several raw receiver gathers, justifying the development of this technology as a surface array for deep targeting. The adopted processing workflow handles noise issues inherent to DAS data and allows us to image the mineralization and surrounding host geological structures including a fault system thought to have played a role in the termination of the deposits at about 1000 m depth.

## Introduction

In recent years, the use of distributed acoustic sensing (DAS) technology for seismic applications for both passive- and active-source methods has increased tremendously. On the active-source methods side, DAS has demonstrated its suitability especially for borehole applications, with vertical seismic profiling (VSP) surveys being well established for reservoir characterization and monitoring of carbon capture and storage sites^1–4^. Some studies have also highlighted the potential of DAS-VSP for mineral exploration purposes^5,6^, although examples of DAS applications in mining and exploration remain relatively limited^7^, particularly using surface arrays for deep mineral deposit imaging. Our study aims to evaluate the applicability of a surface-deployed DAS array for mineral exploration and to assess its performance relative to that of a collocated, simultaneously acquired nodal broadband seismic dataset.

DAS is a technology consisting of measuring strain changes along a fiber-optic cable using phase optical time domain reflectometry^8^. The interrogator unit (IU) sends short laser pulses through the fiber cable and measures phase differences in the backscattered portion of the light travelling along the fiber. Phase changes in Rayleigh scattering between two points separated by the gauge length can be linked to the longitudinal strain exerted on the cable by the seismic wavefield^8,9^. DAS functions as a continuous sensor, providing measurements of dynamic strain rates at closely spaced intervals along the fiber cable. Thus, the fiber cable acts as an array of longitudinally-sensitive (horizontal) single-component relative strain gauges^10^.

Our study utilizes a surface DAS dataset, which was acquired in June 2022 at the Blöberget mine in the Bergslagen mining district in central Sweden. Bergslagen is a well-known mineral province characterized by a wide range of mineral deposits and has historically played a significant role in supporting the Swedish industrial sector^11^. Within this province, the Blötberget deposit comprises high-grade iron-oxides, predominantly magnetite and hematite with localized enrichment in apatite, which in turn also hosts some rare-earth elements (REEs). The ore bodies are hosted in sheet-like horizons ranging from 10 to 30 m in thickness and exhibit a moderate dip of approximately 45° eastward along a NNE-trending zone^12^. Current geological interpretations suggest that the mineralization extends to a depth of 1000 m where a cross-cutting seismic reflector appears to interrupt its continuity^13^ and was suggested to correspond to a fault system.

Mining activities in Blötberget, as in much of Bergslagen, ceased during the late 1970s due to a global decline in iron ore prices. However, renewed interest in the region has emerged due to increasing global demand for raw materials and the recognition of REE potential associated with apatite-bearing iron-oxide deposits as a potential source of extraction. Following the acquisition of a mining concession in 2011, Nordic Iron initiated a comprehensive series of feasibility and exploration studies in collaboration with academic and industrial partners to evaluate the economic viability of the deposit and to further constrain its geological framework^14–16^. Given the depth of the resources in the area (>500 m) and their substantial density and acoustic impedance contrast with the host rocks, reflection seismic methods have proven particularly effective for subsurface imaging in this context. Due to the extensive knowledge acquired from the numerous previous seismic surveys in the area, Blötberget now represents the most extensively seismically investigated mining site in Sweden, serving as a benchmark location for the development and validation of advanced geophysical technologies^17^. As such, it provides an ideal testbed for evaluating the performance of DAS in imaging mineralized systems.

**Fig. 1:**
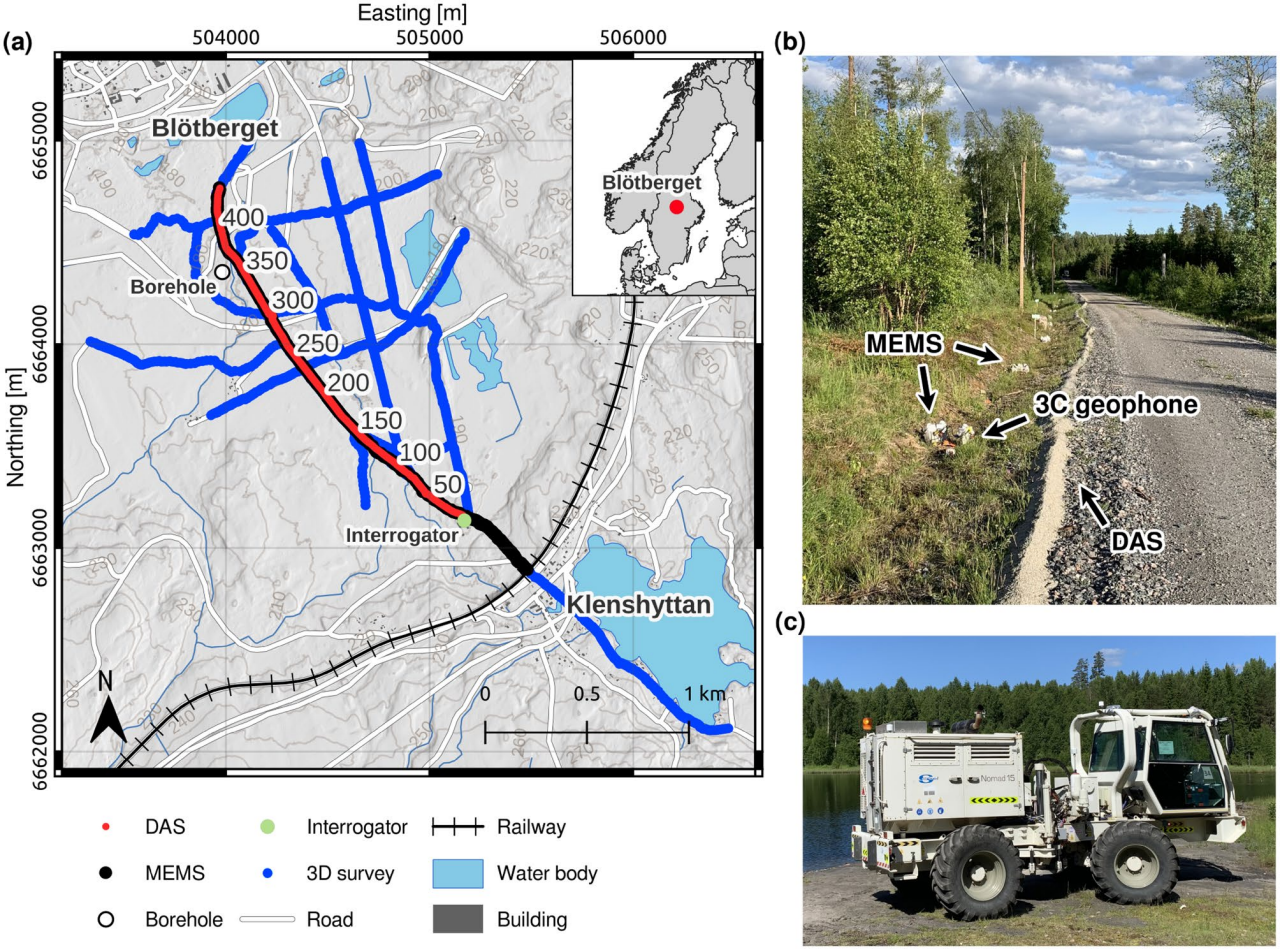
(**a**) The study area and the location of the seismic profile with the surface DAS array in red with the channel numbering. The MEMS-based receivers and shot locations are shown in black and the outline of a previous sparse 3D survey in light blue. Field pictures of the seismic data acquisition with the collocated DAS and MEMS nodal arrays in (**b**) and seismic vibrotruck in (**c**). The elevation data and map features are provided by Lantmäteriet through Uppsala University GET (Geo Extraction Tool) and modified using QGIS V3.22 (https://qgis.org/). Photos were taken by Lea Gyger.

The seismic data used in this study were obtained using a vibrotruck generating a linear sweep of 2–200 Hz. The receiver array was deployed on the downdip of the mineralization and consisted of a 2200 m long straight telecommunication optical fiber cable interrogated with a spatial sampling of 5 m and a 10 m gauge length. The fiber was collocated with 492 micro-electromechanical sensors (MEMS)^18^ and 150 nodal recorders connected to 3-component (3C) geophones^19^ (Fig. 1). The cable was not trenched but covered with gravel by an excavator within a couple of hours to improve its coupling to the ground. Three to ten sweeps were generated at each shot point to increase the signal-to-noise ratio.

The objective of this study is to provide an overview of the quality of the data obtained using the surface-DAS deployment and vibrotruck source in the context of reflection seismology for mineral exploration. We first discuss the data quality and the processing steps necessary to image the mineralization in the unmigrated stacked seismic section. We then qualitatively compare its performance with the MEMS dataset. Finally, we discuss the viability of surface-DAS arrays for mineral exploration.

**Fig. 2:**
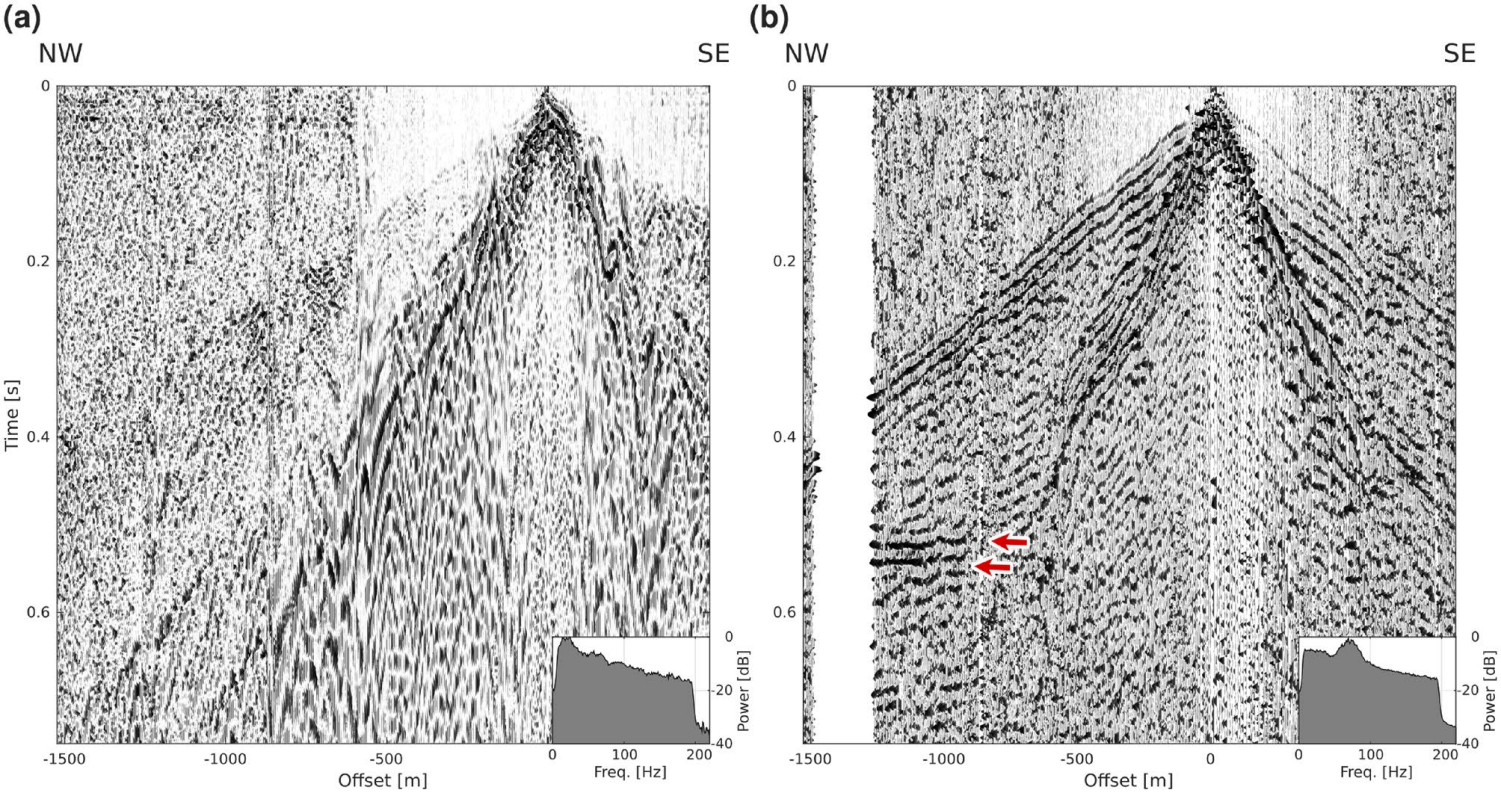
(**a**) An example shot gather after vertically stacking the three repeated shot records showing incoherent noise level across all fiber channels. (**b**) The corresponding receiver gather displaying a much higher signal coherency. The red arrows indicate a clear signature of the reflection from the mineralization. Note that shots were generated further to the south than the length of the fiber cable, leading to more traces being displayed in the receiver gather. The amplitude spectra are shown for the entire gather.

## Data quality

The DAS data were largely contaminated with common-mode noise, appearing as horizontal lines of constant intensity varying from shot to shot. Common-mode noise appears in the data when there is either acoustic noise next to the IU or if the interrogator itself is subject to vibrations^20^. This noise was successfully removed using a horizontal median filter before vertically stacking the shots. Shot gathers, however, are difficult to interpret. Figure 2 presents a comparison between a shot gather and the corresponding receiver gather. Several noteworthy features are apparent. First, a significant amount of noise across the entire dataset can be observed in the shot gather, with a varying frequency content for different channels. While the noise is occasionally coherent across a limited number of channels, its high variability along the seismic profile makes it challenging to track any coherent signal across the gather. The first breaks exhibit weak amplitudes through the whole dataset, and no reflections from the mineralization are discernible.

**Fig. 3:**
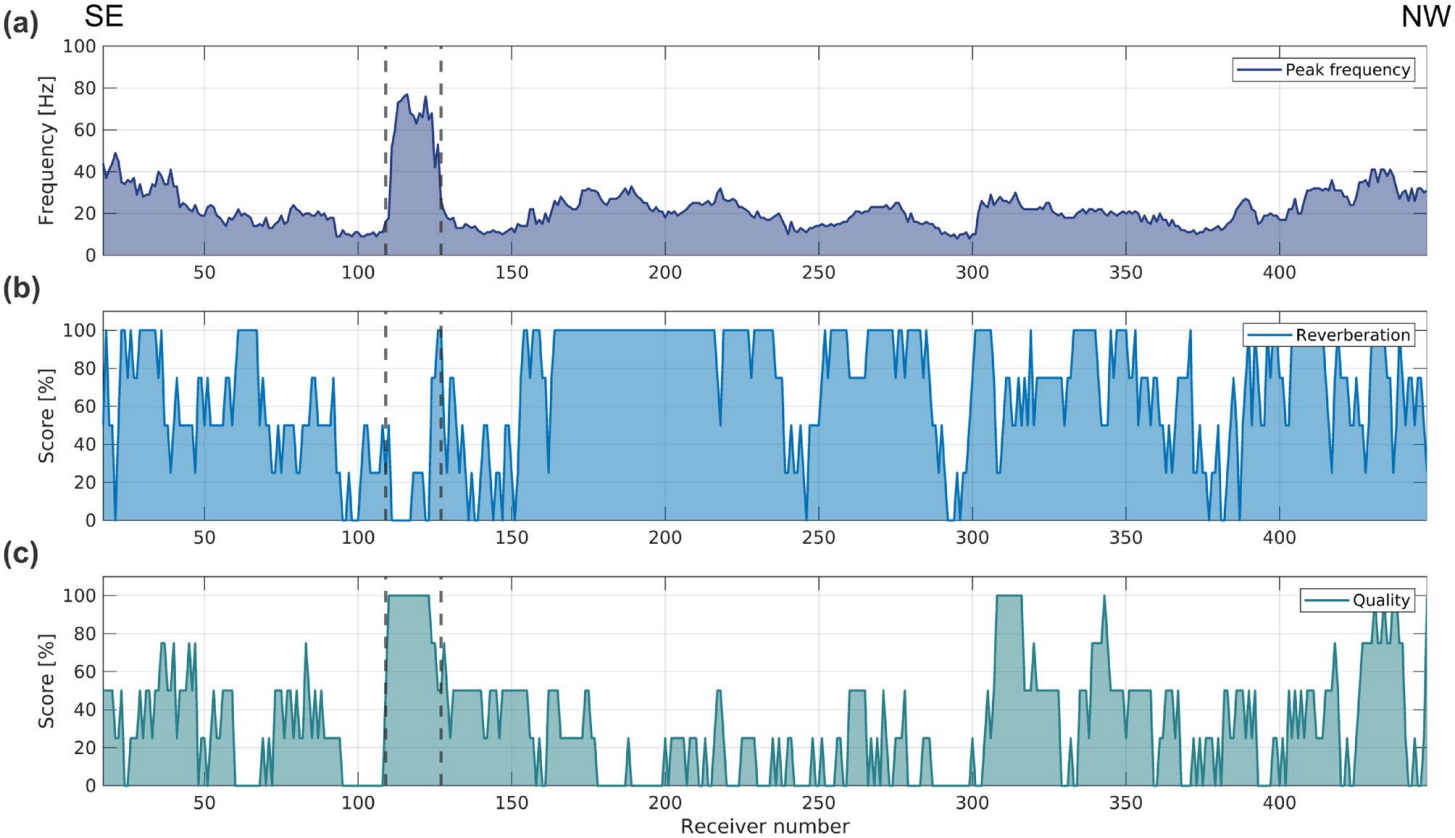
(**a**) Frequency value for the peak amplitude in the amplitude spectra of each receiver gather, (**b**) qualitative assessment of the amount of reverberations and (**c**) qualitative assessment of the data quality for each receiver gather. The vertical dashed lines highlight channels 109-123, which display outstanding data quality compared to the rest of the dataset.

In contrast, the receiver gathers show substantially more coherent energy (Fig. 2b). For high-quality receivers, the first-break signal is traceable across the whole receiver gather and the ground-roll appears continuous. On several receiver gathers, however, the seismic signal is subject to a substantial amount of reverberations, which can occur with the first breaks but are more often appearing with the ground-roll and can propagate up to the end of the 1 s shot record. Whenever these reverberations are present in a receiver gather, they cause a peak in the amplitude spectrum. To characterize these reverberations, we opted for a qualitative assessment of the amount of reverberations and assigned a score to each receiver gather in five increments: 0, 25, 50, 75 and 100% with 0% corresponding to no reverberation and 100% with a maximum amount of reverberation. When comparing the reverberation score to the peak frequency of the amplitude spectrum of each receiver gather (Fig. 3a,b), it appears that the maximum frequency of these reverberations falls within a range of 10–40 Hz. These reverberations are, however, not monochromatic. Their amount, length and peak frequency vary considerably between the receivers but are constant for all shots recorded at one specific receiver, suggesting that they originate mostly from poor coupling between the cable and the ground.

The fiber cable was positioned adjacent to a gravel road running through the forest. Portions of the cable had to be placed on the grass to allow vehicles to drive past, resulting in suboptimal coupling due to the cable hovering slightly above the ground in some areas. The thin gravel cover might not have been sufficient in this instance to mitigate this effect, potentially explaining the observed high variability in the signal-to-noise ratio throughout the dataset. In comparison, the data from the collocated MEMS displayed a uniform quality with a clear first-break signal across all receivers for most of the shots^18^. A detailed comparison by Wilczynski^21^ of the amplitude and phase responses for all three receiver types used in this survey (MEMS, geophones and surface DAS) also suggests that most of the inconsistencies in signal quality along the DAS cable is attributed to coupling issues between the fiber-optic cable and the ground. Our goal, however, was to check if this DAS setup can be useful without causing any damage to the road.

In the receiver gathers where a reflection from the mineralization is clearly visible, most of the signal from the mineralization falls into the 50-90 Hz range, a much narrower bandwidth than the original sweep. While a wider range of useful frequencies was contained in the rest of the dataset, the noise levels in both the low and high-frequency ends were sufficiently high to necessitate their exclusion. Finally, extensive trace editing was required to image the mineralization reflection on the unmigrated stacked section and this task could only be performed by examining the receiver gathers.

## Results

**Fig. 4:**
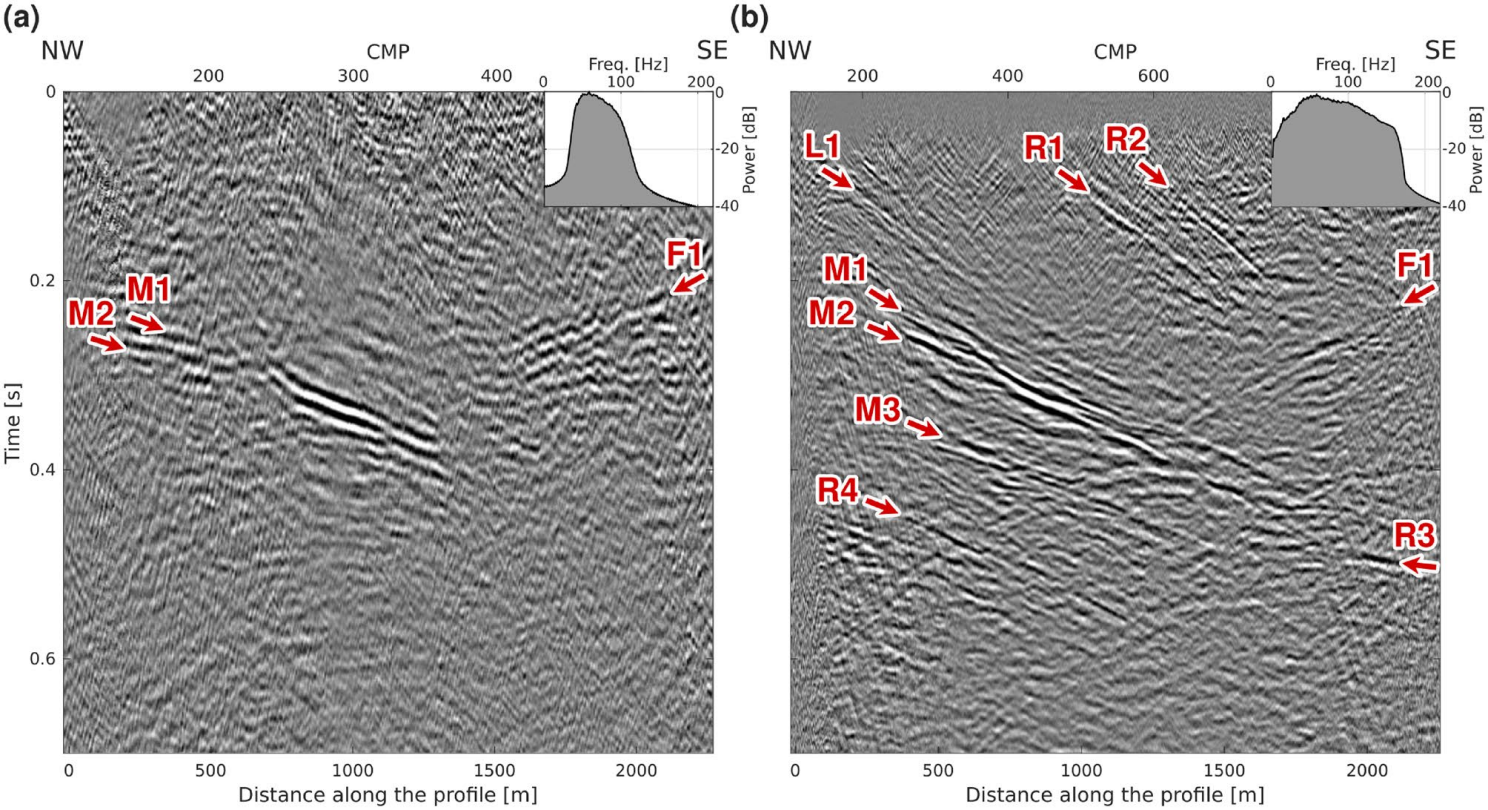
(**a**) Unmigrated stack from the surface DAS array and (**b**) corresponding section from the MEMS nodal array showing much broader bandwidth and therefore higher resolution. The labels indicate the main reflections in each section.

Figure 4a displays the unmigrated stacked section of the DAS data. Clear reflections from the mineralization (M1-M2) can be followed down to approximately 0.4 s, although lacking continuity between CMP 200 and 220. A cross-cutting reflection with an opposite dip (F1) to the mineralization is also visible despite being highly fragmented. This reflection has been interpreted to originate from a fault in previous studies^13,18,22^. Refraction and residual static corrections greatly improved the continuity of the reflection from the mineralization, and were necessary to make the fault reflection appear in the stacked section. However, our refraction static corrections might not have been the most accurate due to the sparsity of good quality first breaks in the DAS data.

The unmigrated stacked section of the MEMS nodal data from the same survey^18^ is shown in Fig. 4b for comparison. The MEMS data were processed with the aim of retaining the entire bandwidth of the source signal, which explains the difference in frequency content between the two datasets. L1 corresponds to a lithological contact between intrusive and volcanic rocks when projected to the surface^13,23^. The origin of R1-R4 remains unknown but R1 and R2 are located close to another lithological contact and a region of weak magnetic anomaly^18^. It is interesting to note that all these reflections (L1, M3, R1-R2, R4), although having a similar dip to the mineralization, are not resolved on the DAS data. One potential explanation for the absence of these reflections can be the limited frequency bandwidth of the data, which may be insufficient for imaging thin geological features and features characterized by low contrast. We applied a bandpass of 30-50-90-135 Hz on the data. With the vertical seismic resolution generally corresponding to a quarter of the dominant wavelength, we could achieve a resolution of 15 m for a 90 Hz signal assuming a bedrock velocity of 5600 m/s. In comparison, we were able to reach a vertical resolution of 7 m with the MEMS data with a signal of 180 Hz for the same velocity. Additionally, the elevated noise levels observed at short travel times may further impede the imaging of reflections R1–R2 within this low-fold time interval.

**Fig. 5:**
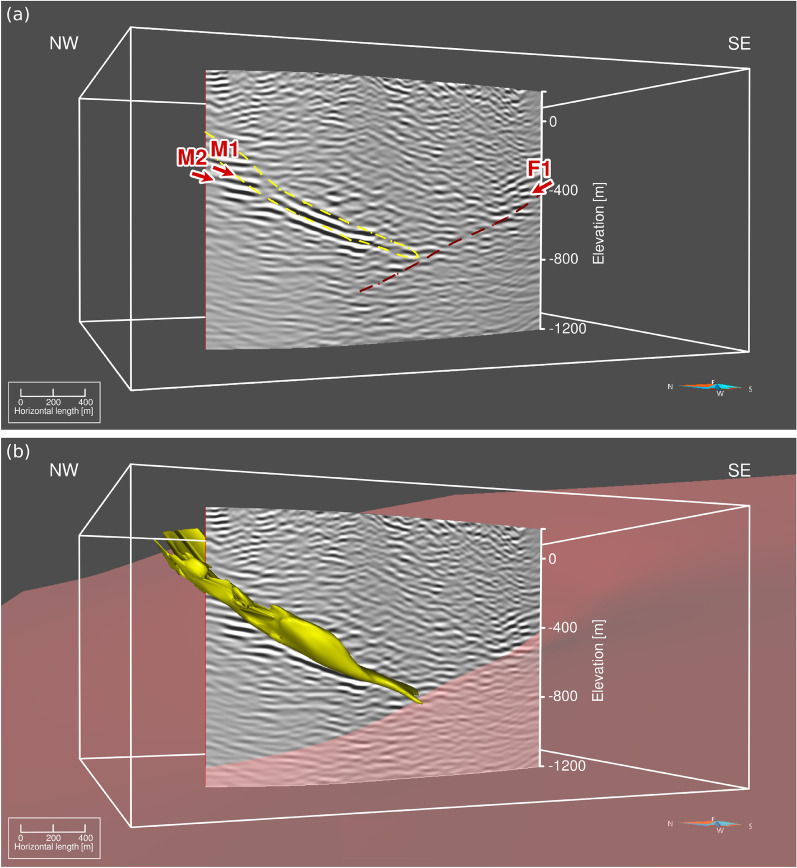
(**a**) Migrated stacked section with the outline of the mineralization in yellow and the cross-cutting fault in red. (**b**) Same section with the 3D surface of the mineralization and the cross-cutting fault plane. Overall, the geometry of the DAS migrated cross-section shows a good correlation with the modeled ore-body. The labels indicate the mineralization (M1-M2) and interpreted cross-cutting fault (F1). Aspen SKUA V15(5.0.0.0) software (https://www.aspentech.com/en/products/sse/aspen-skua) was used for 3D visualization and preparation of the figure in this study.

**Fig. 6:**
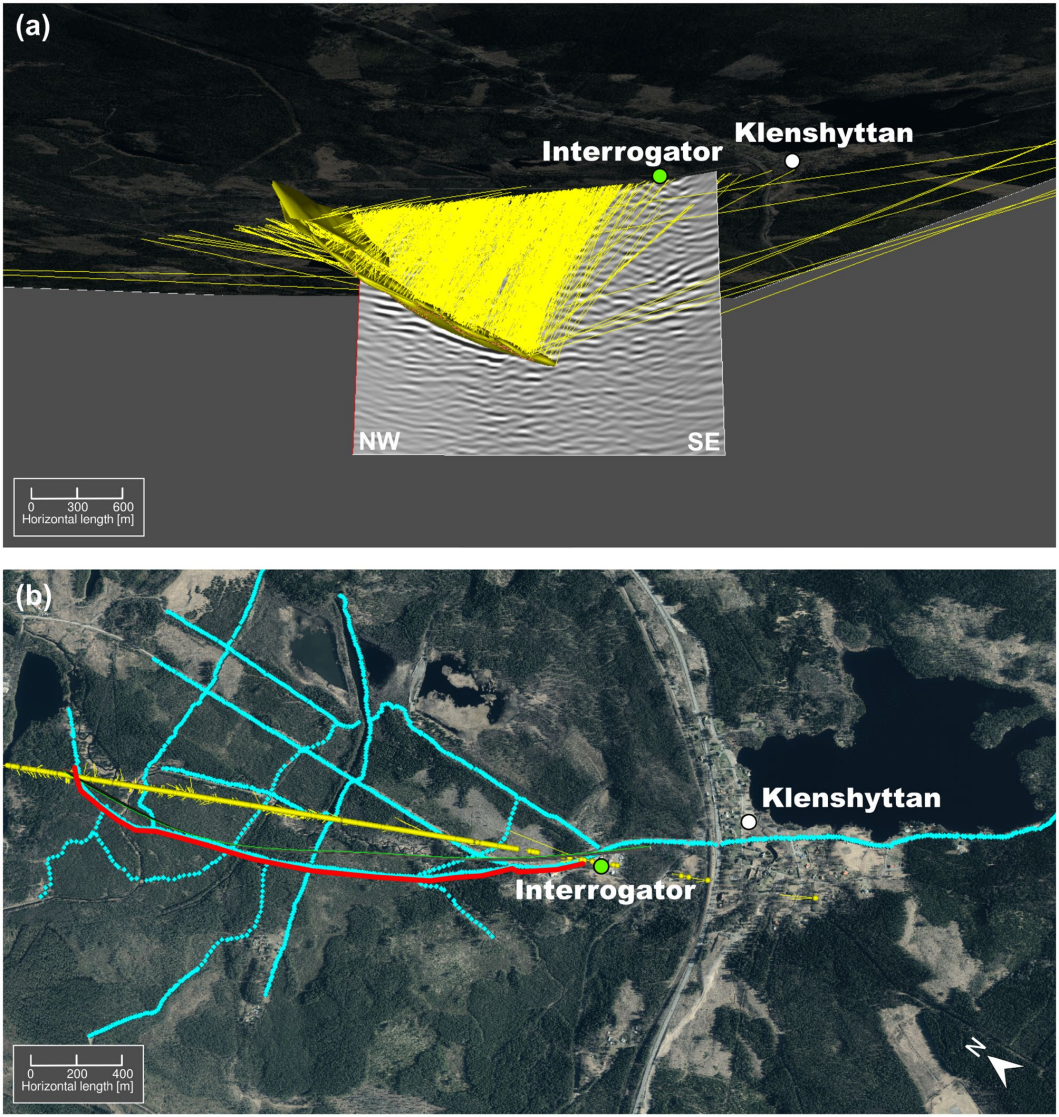
(**a**) 3D view of the mineralization (yellow surface) with the rays of an exploding reflector model to depict the contribution of the reflected rays to the surface receivers. The migrated seismic section is displayed in the background. (**b**) Top view of the incoming rays on the surface shown by yellow dots. The red line corresponds to the location of the fiber-optic cable and the blue points correspond to the receiver array of the sparse 3D survey that led to modeling the 3D surface of the mineralization shown in (**a**). This exploding reflector model shows that the contribution of the receiver south of the interrogator play little role in imaging the deeper part of the mineralization. The aerial photo is provided as raster image by Lantmäteriet GSD-Orthofoto 1 m through Uppsala University GET (Geo Extraction Tool). Aspen SKUA V15(5.0.0.0) software (https://www.aspentech.com/en/products/sse/aspen-skua) was used for 3D visualization and preparation of the figure in this study.

After migrating the seismic section in the time domain, the geometry of the deposit aligns well with the modeled 3D surface of the ore for the central portion of the reflection (Fig. 5). This 3D surface was built based on borehole data and the results from a sparse 3D seismic survey from 2019^13^, the outline of which are shown in Fig. 1a. The upper parts of M1-M2 exhibit a slightly more horizontal orientation than in the previous studies. The cross-cutting fault (F1) shows poor continuity, likely due to the high noise level remaining in the data. The reflection associated with the mineralization (M1-M2) does not fully match the depth extent of the modeled ore body from the 3D seismic survey in the area. In order to understand if this lack of continuity at depth is dependent on the DAS cable being shorter (and having a lower fold) than the 2019 sparse 3D and 2022 2D receiver arrays, we calculated the ray paths reaching the surface along the seismic profiles using the modeled 3D surface of the mineralization as an exploding reflector (Fig.  6a). Each point on the 3D surface of the mineralization generates a ray perpendicular to it. For visualization purposes, the rays are limited to the ones traveling upwards and reaching a line drawn at the surface corresponding approximately to the location of the 2D MEMS profile (Fig. 6b). According to these results, the receivers located south of the interrogator contribute very little to image the deepest part of the mineralization. This lack of continuity should therefore rather be attributed to poor cable coupling rather than to the survey layout only.

## Surface DAS for mineral exploration

**Fig. 7:**
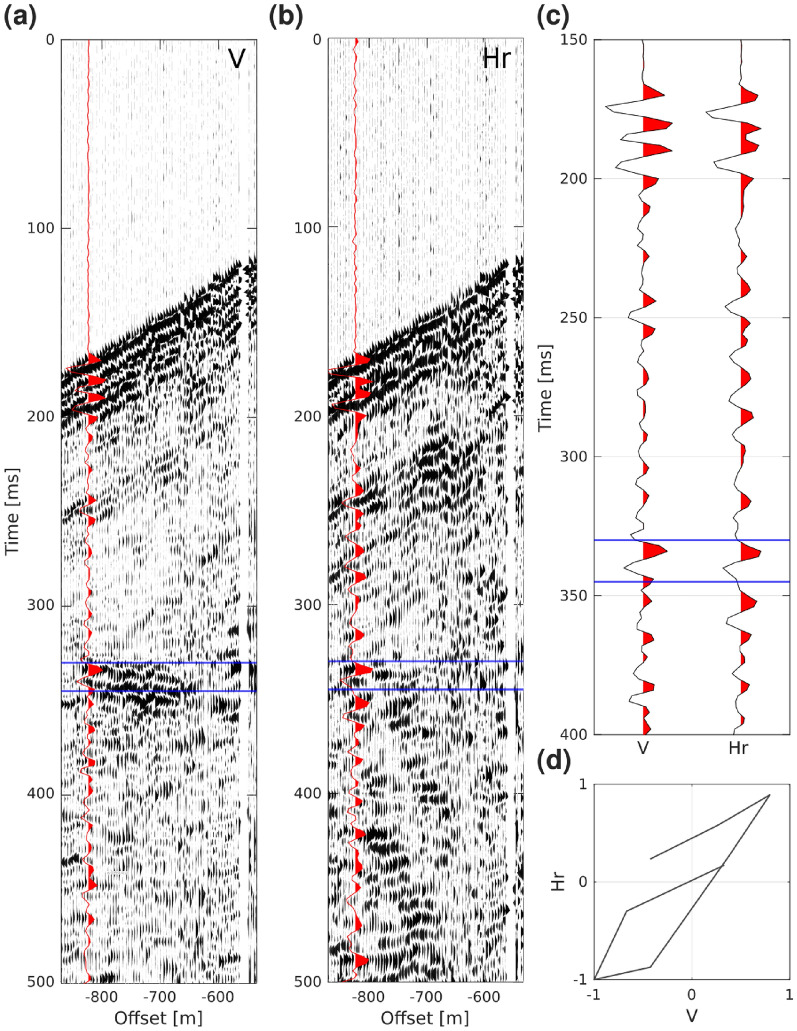
Partial receiver gathers of the (**a**) vertical (V) and (**b**) horizontal radial components (Hr) of a 3C geophone with a sampling rate of 2 ms. During the acquisition, the radial component was aligned with the fiber-optic cable. The red traces correspond to the same shot record. The blue lines highlight the reflection from the mineralization and define the time window used for the hodogram. (**c**) A close-up window of the two traces used to compute the hodogram presented in (**d**), with the wavefield reflected on the mineralization shows both energy in the vertical and radial components.

Despite the many successful applications of DAS as a surface array for earthquake detection and as a borehole array for monitoring and VSP surveys, there are to date and to our knowledge no studies showing the successful application of a surface DAS array for direct deposit targeting. There are several issues to overcome compared to the use of single point MEMS- or geophone-based receivers: one of the biggest being the directional sensitivity of the fiber-optic cable. The directional sensitivity is proportional to $cos^2\theta$, with $\theta$ being the angle between the incident wave and the cable itself^9^. This angular sensitivity allows for picking up reflections from dipping targets, but inhibits recording reflections from sub-horizontal targets at close offsets. The P- and S-wave sensitivity is broadly discussed by Martin et al.^24^. In the case of sub-horizontal targets, the use of a helically wound DAS cable instead of a straight fiber-optic cable could be more beneficial but considering their current price, they remain cost-ineffective for surface DAS arrays. In our study, the fiber sensitivity likely worked in favor of our dipping target. With our seismic source primarily generating vertical P-waves and the mineralization being inclined at an angle of approximately 45°, there is a clear horizontal component of the wavefield reflected off the mineralization. Figure 7 displays the hodogram analysis of the vertical and radial component of a 3C geophone-based recorder, focusing around the reflection from the mineralization. The geophone was oriented with the radial component being parallel to the surface DAS cable. With these favorable settings, we were able to record the horizontal component of the wavefield reflected from the mineralization with a straight fiber-optic cable lying on the ground with a minimal coupling strategy.

One downside of DAS systems is the high level of noise recorded. Our acquisition could have been improved in this regard in several ways. The interrogator could potentially have been better isolated from the surrounding noise to limit the amount of common-mode noise, although it can be challenging to be both close to a power source while simultaneously away from anthropogenic noise. Several studies have highlighted the importance of coupling^25,26^ to improve the signal-to-noise ratio and preserve amplitudes. There are, however, few studies investigating how to quantify the quality of the coupling between the DAS cable and the ground once the data acquisition in completed. We applied the coupling coherency quantification developed by Hudson et al.^26^ on our data to provide an objective measure of the coupling and hence data quality along the cable. This method consists in measuring the relative coupling of the cable by assessing how the seismic trace of one channel along the cable is similar to the previous one. This coherency measurement is done by multiplying each time sample of these two traces and dividing the result by their absolute product, yielding a coherency value of either 1 or $-1$; 1 if the amplitudes of both traces are on the same side of the zero axis, $-1$ otherwise. The mean is then taken for the time window selected, resulting in one value per trace and per gather. Unfortunately, this method did not perform well on our dataset and did not reflect the actual quality of the coupling. Twelve channels along the DAS cable (channel 109-123) display an outstanding quality level compared to the rest of the dataset. These were not picked up by the coherency method, firstly because this method only provide relative measurements to the neighboring channel but mostly due to the presence of reverberations as mentioned above. Many channels along the cable are subject to a large amount of reverberations, which vary in strength and frequency. Because these reverberations are channel specific, they do not start at the same time sample and do not have the same period across neighboring traces for the same shot gather. Therefore, the coherency will always be low.

Another way of assessing the coupling and data quality would be to calculate the signal-to-noise ratio (S/N) of a window above the first breaks containing only noise and a window centered around a clear signal, such as the first breaks. We refrained from using this method for the following reasons: (1) since the DAS cable acts as a horizontal sensor rather than a vertical one, the first-break signal can be quite weak and is therefore not representative of the rest of the gather, (2) if present in the receiver gather, reverberation effects can give the illusion of a strong first-break signal, when this periodic signal is in fact contaminating the whole gather and contributing to additional noise in the dataset. We therefore opted again for a qualitative approach to assess the data quality of the receiver gather and therefore of the cable coupling. Each receiver gather was assigned a score in five increments: 0, 25, 50, 75 and 100%, with 0% corresponding to very poor data quality where the first breaks cannot be distinguished, a large amount of noise is present and no reflection from the mineralization is visible. On the other hand, 100% corresponds to a good quality gather, with first breaks present at all offsets and where we observe a clear reflection from the mineralization. The results are presented in Fig. 3c. Although it is true for most receivers that a high amount of reverberation corresponds to low quality data, it is not always the case, as can be seen for channels 307-320.

A numerical study by Celli et al.^25^ modeled the DAS cable response to a seismic source and analyzed the coupling and site effects on the strain recorded. According to the results, the stiffness of the surrounding material has the largest distortion impact on the recorded strain, with low stiffness causing amplification and phase delays, especially for surface waves. Additionally, placing the source on top of the cable instead of 500 m away and below the cable, led to additional amplification, distortion and phase delay of the surface waves. Hence, apart from the possible poor coupling with the ground due to the presence of grass or tension on the cable causing it to vibrate, the loose gravel cover might have partly contributed in generating and sustaining the reverberations observed in our dataset. A feature standing out the data displayed in Fig. 3 is the high peak frequency, low reverberation amount and high quality of channels 109-123. These channels are located along the forest path about 120 m north of the crossing with the main road. There, the path crosses an outcrop, corresponding to the absence of a low velocity surface layer, which in turn could explain the limited amount of ground roll, low frequencies and signal distortion. Future surface DAS surveys could benefit from accurately recording the ground conditions in order to fully understand how varying coupling conditions influence the data quality.

Trenching the fiber would likely have provided higher data quality. With the purpose of mineral exploration in mind, we were, however, interested in rapid and simple deployments. Three people only were needed to deploy the cable along the road, one person driving the car where the cable drum was placed on a rotating table and the other two laying the cable on the ground. Finally, a contractor driving an excavator covered the whole fiber with a layer of gravel. The entire cable deployment only took a few hours. The results of this study are also relevant for areas where it is impossible to trench the fiber and opens the possibility for reflection seismic studies using existing fiber networks and plugging into dark fiber cables. Further advantages of using surface DAS with the approach taken in this study are: the removal of pre-deployment charging of the receiver units and post-acquisition data harvesting, the possibility of high spatial sampling that allows for unaliased recording of the seismic wavefield, and the flexibility in array length, which can be extended up to several tens of kilometers. The fiber-optic cable was easily removed, leaving almost no footprints from the survey apart from the light gravel cover on the side of the road.

The significant levels of noise observed both in the low- and high-frequency range of the DAS dataset led to a reduced usable frequency bandwidth. However, depending on the purpose of the survey, the DAS system can still yield satisfactory results. Despite the narrower bandwidth and the apparent low data quality, we successfully imaged the mineralization and the cross-cutting fault using the surface DAS array. Furthermore, based on the recorded signal, the sweep frequencies could be optimized to better match the sensing range of the DAS system, potentially increasing productivity during the data acquisition. Given that DAS is a distributed sensor and therefore allows for a dense spatial sampling along the fiber, we could have maximized this feature and used a channel spacing of 1 m instead of 5 m. This adjustment would have allowed for binning more data, therefore improving the signal-to-noise ratio for a CMP spacing of 5 m. These improvements can be readily applied in future surveys.

**Table 1:** Data acquisition parameters (June 2022).

**Receivers**	DAS	MEMS	3C geophone
Profile length	2200 m	2500 m	1500 m
Number of receivers	448 measurement points	492	150
Spacing	5 m, with 10 m gauge length	5 m	10 m
Sampling frequency	20 kHz	1 kHz	500 Hz
**Source**			
Vibrator truck	77 kN peak force		
Shot spacing	5 m		
Sweep	2–220 Hz, linear, 18 s		
	3–10 sweeps/point		

## Conclusions

We have demonstrated that a surface DAS array can be used to image not only an iron-oxide deposit but also key geological features in the host rock, such as a fault cross-cutting the mineralization. Due to the high variability in noise levels along the cable, the use of receiver gathers proved essential in generating an adequate processing sequence and in identifying and discarding the poor quality part of the data. It is essential that both domains of the data are scrutinized during the data quality assessment stage. Most of the noise present in this dataset is likely related to the coupling issues. Key processing steps including the removal of common-mode noise prior to vertically stacking the repeated shot records, the application of a narrow bandpass, trace editing as well as refraction and residual static corrections were critical in achieving a successful final seismic section that effectively imaged the mineralization. Although the data quality is significantly lower than that of the MEMS, we are able to image the ore body and important host-rock structures, such as a major reflector cross-cutting the mineralization. These results are promising, and under appropriate acquisition conditions, considering target geometry and desired resolution, we believe surface DAS has substantial potential for reflection seismic applications and could serve as a viable alternative to conventional single-point recorders used in mineral exploration.

## Methods

### Data acquisition

The surface DAS dataset was acquired in June 2022 in Blötberget as part of a pilot study experimenting with several receiver arrays deployed above the dipping mineralization^18^. The surface DAS array consisted of a 2.2 km-long fiber-optic cable partly co-located with 492 MEMS-based receivers (5 m spacing, 1 ms sampling rate) and 150-3C geophones (10 m spacing, 2 ms sampling rate). We used a DAS interrogator in combination with a straight, telecommunication fiber-optic cable. The IU was placed in a cabin a few tens of meters away from the acquisition profile (Fig. 1a). The spatial location of the channels along the fiber was done through a tap test consisting of 17 points combined with DGPS (differential global positioning system) measurements. The remaining channel locations were assigned by interpolating between the tap test points. The data were recorded as differential phase at a frequency of 20 kHz. The channel spacing was set to 5 m, resulting in 448 channels evenly spread along the cable with a gauge length selected of 10 m. We used a seismic vibrotruck with a peak force of 77 kN as our vertical seismic source. The source performed three sweeps at every MEMS node location (5 m spacing). The number of sweeps were increased up to ten in the vicinity of a borehole as part of a walkaway VSP DAS experiment^18^. The sweeps had a duration of 18 s, with a frequency linearly increasing from 2 to 200 Hz at 70% of the vibrotruck’s peak force. The key acquisition parameters are summarized in Table 1.

### Data processing

We started by extracting the shot gathers from the continuous recordings. An issue in the GPS timing of the interrogator caused a time delay in the DAS data of about 500 ms. The DAS data were converted from phase to strain rate and then decimated to 1 kHz. We then proceeded to cross-correlate the data with the theoretical sweep from the vibrotruck. The data were quite heavily contaminated with common-mode noise manifesting itself by horizontal lines across the shot gathers, varying in time and amplitude for every shot. Some of this noise had already been removed by the sweep cross-correlation. The rest was removed using a horizontal median filter with a window of 431 traces. The repeated shot records were then vertically stacked to improve the signal-to-noise ratio. Although the sweeps were performed in a frequency range of 2–200 Hz, the useful bandwidth of the DAS data was situated between 50 and 90 Hz. A 30-50-90-135 Hz bandpass filter was used on the entire dataset (Supplementary Fig. S1).

Both the signal from the first breaks and the reflection from the mineralization showed some reverberation effects. These effects were however successfully compensated for by bandpass filtering the data and using pre- and post-stack gapped deconvolution filters. A pre-stack deconvolution filter with a filter length of 100 and 28 ms gap was used to compress the signal from the first breaks and reflection from the mineralization (Fig. S1). The processing sequence was then followed by first-break picking and consequent trace editing. The refraction static corrections were computed using the first break picks and applied on the data along with the elevation static corrections. The first-break signal and ground roll were removed using two separate median filters with a constant velocity of 5400 m/s and 2800 m/s respectively. We used a top-mute to remove the noise above the first breaks and proceeded to two rounds of velocity analysis combined with residual static calculations (Fig. S1). Applying the normal-moveout corrections (NMO) from our velocity analysis and an automatic gain control (AGC) window of 300 ms concluded our pre-stack processing sequence. The data were subsequently stacked to a 2D seismic section. Post-stack processing involved the use of FX-deconvolution and trace balance to improve the coherence of the reflections. Finally, the data were migrated in the time domain using a finite-difference migration algorithm. The velocity model used was based on a 1D velocity model extracted from the borehole DAS recording close to the seismic profile and reported in^18^. Supplementary Table S1 presents a summary of the processing parameters.

## Supplementary Information

Supplementary Information 1.

Supplementary Information 2.

## Data Availability

The data that support the findings of this study are available from the corresponding author upon request.
