# Supplementary material for: Surface distributed acoustic sensing for mineral exploration
Source: Sci Rep. 2025 Dec 9;15:43391. doi: 10.1038/s41598-025-29964-6 (PMC12689791; doi:10.1038/s41598-025-29964-6)
Supplement: Supplementary file 1 — Supplementary Information 1. [file 41598_2025_29964_MOESM1_ESM.pdf]

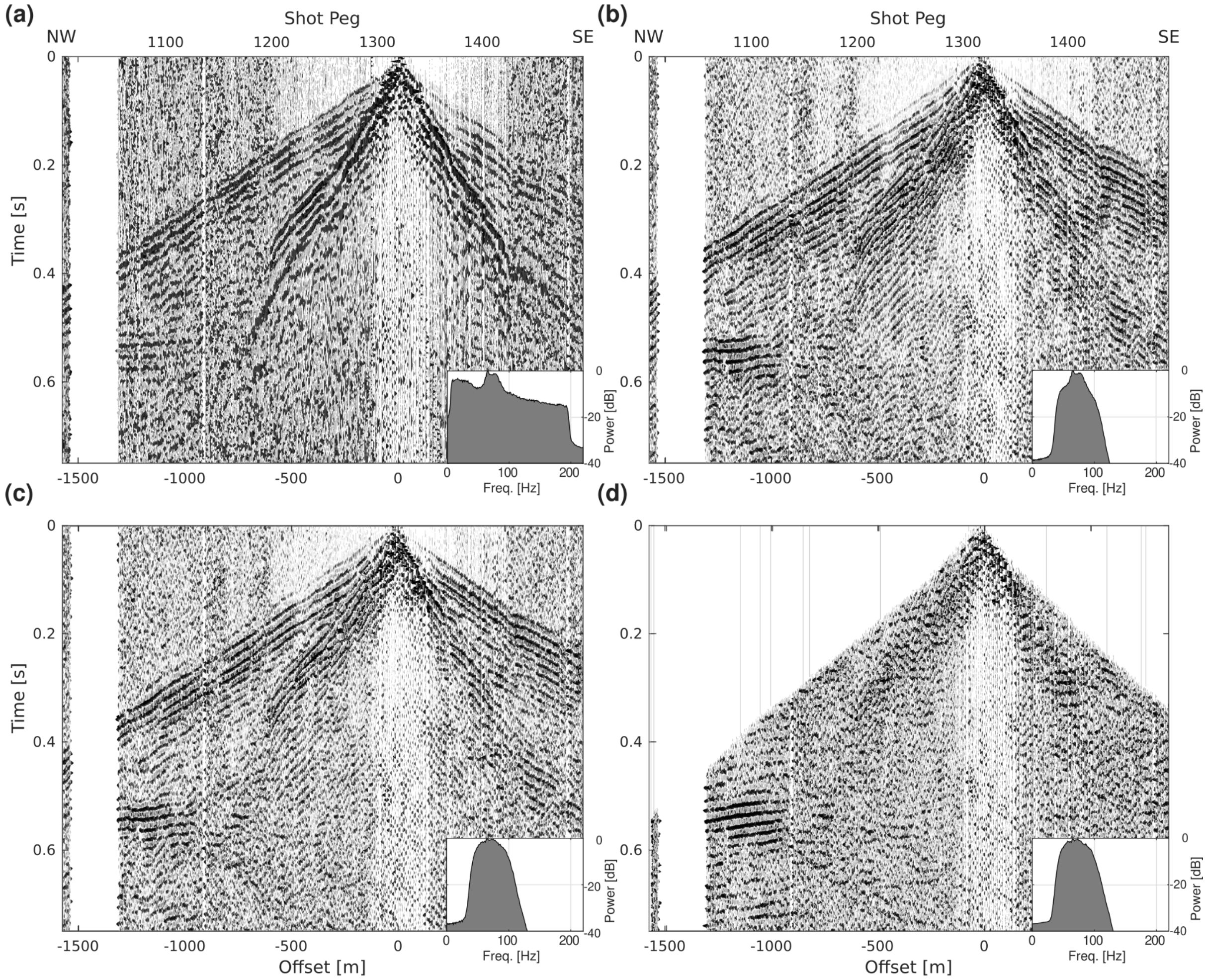

Fig. S1 (a) A high-quality receiver gather after removing the common-mode noise and vertically stacking the repeated shot records, (b) after applying refraction static corrections and bandpass filtering the data (30-50-90-135 Hz), (c) after applying pre-stack Wiener gapped deconvolution and (d) after removing the ground roll, applying a top mute and residual static corrections. The amplitude spectra are shown for the entire gather.
