# Supplementary material for: Surface distributed acoustic sensing for mineral exploration
Source: Sci Rep. 2025 Dec 9;15:43391. doi: 10.1038/s41598-025-29964-6 (PMC12689791; doi:10.1038/s41598-025-29964-6)
Supplement: Supplementary file 2 — Supplementary Information 2. [file 41598_2025_29964_MOESM2_ESM.docx]

**Table S1.** Processing sequence applied to the DAS dataset

| **Step** | **Process** |
| --- | --- |
| 1 | Interrogator noise removal with horizontal median filter |
| 2 | Vertical stack of repeated shots |
| 3 | Bandpass filter (30-50-90-135 Hz) |
| 4 | Pre-stack Wiener deconvolution (gap: 28 ms, length: 200 ms) |
| 5 | First-break picking |
| 6 | Refraction and elevation statics |
| 7 | Trace editing |
| 8 | Median filter to remove the first-breaks (5350 m/s) |
| 9 | Median filter to remove S- and surface-waves (2800 m/s) |
| 10 | Velocity analysis and residual static corrections (2x) |
| 11 | AGC (window length: 300 ms) |
| 12 | Stack (5 m CMP spacing) |
| 13 | FX-deconvolution (Filter length: 19 traces, 100 ms) |
| 14 | Trace balance |
| 15 | Finite-difference post-stack time migration using 1D velocity model from borehole DAS |
| 16 | Post-stack Wiener deconvolution (gap: 17 ms, length: 100 ms) |
| 17 | Bandpass filter (30-50-90-135 Hz) |
